# Detection of sleep apnea by case-finding and home monitoring with Somnolter^®^: a pilot study

**DOI:** 10.1186/1756-0500-7-616

**Published:** 2014-09-08

**Authors:** Fabienne Cattrysse, Mathias Peeters, Sanne Calaerts, Karen Ferson, Jean-Marie Degryse

**Affiliations:** Department of Public Health and Primary Care, Katholieke Universiteit Leuven (KUL), Leuven, Belgium; Institut de Recherche Santé et Société, Université Catholique de Louvain (UCL), Brussels, Belgium

**Keywords:** Sleep apnea, Somnolter^®^, OSAHS, OSAS, AHI

## Abstract

**Background:**

Obstructive sleep apnea and hypopnea syndrome (OSAHS) is a disorder that causes clinical symptoms (e.g. snoring, excessive daytime sleepiness and impaired concentration) that may increase the risk of traffic accidents, cardiovascular disease, type 2 diabetes and reduce the quality of life. A recently developed device (Somnolter^®^) detects apneas and hypopneas in a home setting, allowing to detect OSAHS in a more comfortable environment compared to the gold standard polysomnography. The aim of our study was to investigate whether the Somnolter^®^ is useful in family practice to identify patients with OSAHS.

**Methods:**

Questionnaires were offered to patients in five general practitioner (GP) practices.

Based on the questionnaire and body mass index, patients with an increased risk of OSAHS were contacted to collaborate in the study. In this convenience sample, 18 patients were successfully tested with the Somnolter^®^ measuring SaO_2_, mandibular movements, body position, heart rate, nasal air flow and thoracic and abdominal breathing movements. The Somnolter^®^ automatically analyses the data and different parameters to detect OSAHS. Afterwards, the data were manually revised by the researchers.

**Results:**

Out of 365 subjects, 31 met the inclusion criteria and 18 were successfully tested at home. Sixteen out of 18 patients had an Apnea Hypopnea Index (AHI) ≥ 5, ten of them had mild OSAHS, 3 were categorized as moderate OSAHS and finally 3 matched the criteria of severe OSAHS.

**Conclusion:**

The proposed case-finding strategy still needs optimization, but is considered helpful in selecting patients at high risk of OSAHS. OSAHS was detected in 14 out of 18 patients tested with the Somnolter^®^. In the future the Somnolter^®^ might be a feasible alternative to diagnose OSAHS.

**Electronic supplementary material:**

The online version of this article (doi:10.1186/1756-0500-7-616) contains supplementary material, which is available to authorized users.

## Background

Sleep apnea is a pathological phenomenon which can result in clinical symptoms, e.g. daytime fatigue, excessive sleepiness, impaired concentration, depression. The combination of symptoms and proven breathing abnormalities during polysomnography (PSG) is historically framed in more or less distinguished syndromes. The obstructive sleep apnea syndrome (OSAS) is originally described by Gulleminault in 1976 [1]. When not only apneas but also hypopneas are taken into account, the name changes into obstructive sleep apnea hypopnea syndrome (OSAHS). Also central sleep apnea-hypopnea syndrome (CHAHS), Cheyne-stokes breathing syndrome (CSBS) and sleep hypoventilation syndrome (SVHS) are recognized as pathological entities. The upper airway resistance syndrome (UARS) is a more controversial term that covers the part of the spectrum without oxygen desaturation.

Of these sleep related breathing disorders, OSAHS is the most common worldwide [2]. Despite the high prevalence, 70-80% of patients with OSAHS remains undiagnosed [3–5]. Numerous studies show prevalence rates of 4% in men and 2% in women [6, 7] while other large population studies reveal increased apnea-hypopnea indexes (AHI) in up to 26% of participants [6, 8–10]. These prevalence rates differ substantially because of inadequate syndrome definitions leading to difficulties to correctly compare results. Also in children OSAHS is common. A literature review of Marcus et al. demonstrates that up to 5.7% of the screened pediatric population has OSAHS [11]. Therefore, even children and adolescents with a high risk of having OSAHS, should be screened. Important studies concerning the prevalence of OSAHS are mentioned in Additional file 1: Appendix 1.

In 1999 the American Academy of Sleep Medicine (AASM) Task Force developed recommendations for OSAHS syndrome definition and diagnostic measurement techniques [12]. These diagnostic criteria were established to minimalize the difficulties of comparing study results, e.g. prevalence rates. Diagnostic criteria are cited in Additional file 1: Appendix 2. The purview and limitations of these criteria are further explained in Additional file 1: Appendix 3.

It is crucial for health care professionals, especially when working in primary care, to gain the necessary knowledge about risk factors for OSAHS in order to adequately identify high risk patients. Both modifiable and non-modifiable risk factors associated with OSAHS are summarized in Additional file 1: Appendix 4. From all risk factors, excess body weight seems to be one of the most important risk factors for OSAHS [5, 13, 14]. Therefore long-term weight reduction might be a helpful factor in the conservative treatment of obstructive sleep apnea.

OSAHS is also associated with a number of negative consequences on health. Studies show an increased risk of traffic accidents, arterial hypertension, stroke, depression, cardiovascular diseases and type 2 diabetes [15]. The associated morbidity and its strength of evidence is described in Additional file 1: Appendix 5 [16–18]. Some inconsistency about the association between OSAHS and coronary artery disease, arrhythmias, heart failure, type 2 diabetes and depression remains. On the other hand a remarkable independent association with arterial hypertension, stroke, cardiovascular mortality and death from all-causes is clearly proven. Some of the underlying pathophysiological mechanisms are described in Additional file 1: Appendix 6.

**Figure 1:**
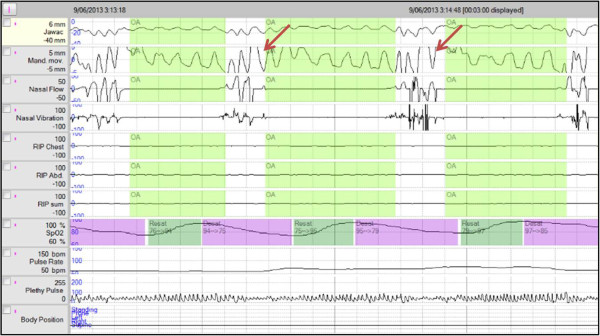
**Mandibular movements analysed by the JAWAC-sensors (red arrows).**

The aim of this pilot study was to set up a case-finding strategy to identify high risk patients in whom it could be useful to screen for OSAHS as it is a highly prevalent disorder with possible dangerous sequelae and very effective treatment options (education and continuous positive airway pressure) [19]. In a selected number of high risk patients, a recently developed device, the Somnolter^®^, was then used to investigate whether obstructive sleep apnea could be detected. The Somnolter^®^ is proven by Cheliout-Heraut et al. in 2011 to be a valuable alternative with a similar sensitivity and specificity as an in-hospital PSG, the current gold standard, for diagnosing sleep apnea syndrome [20, 21]. Both PSG and the Somnolter^®^ record the traditional parameters essential for the diagnosis of sleep apnea syndrome but only the Somnolter^®^ additionally records mandibular movements (Figure 1). These movements represent the respiratory effort during sleep and will reflect the number of apnea episodes which are very important for the diagnosis of sleep apnea syndrome. The mandibular movements also allow to assess the total sleeping time. Together with the other features of the device, the recording of mandibular movements make the Somnolter^®^ feasible to detect sleep apnea in a comfortable environment, such as the patients home.

## Methods

### Sampling

In this convenience sample, patients were selected using a questionnaire based on recent literature on OSAHS and the Epworth Sleepiness Scale (ESS). During five consecutive workdays, Dutch and French brochures containing a questionnaire were offered to patients in four Flemish general practitioners (GP) practices and one mixed Flemish and French speaking GPs practice. These questionnaires asked for the patients name, date of birth, sex, weight, height, an answer on three screening questions and the ESS. The three screening questions enclosed : 1) ‘*Do you snore or did someone ever tell you that you snore?’,* 2) ‘*Do you feel extremely tired at daytime?’* and 3) ‘*Do you experience difficulties to concentrate at daytime?’*. In three practices, the brochures were systematically handed out to all patients aged 25 and older. In the other two practices the brochures were offered to the patients by placing them in the waiting room. All brochures were collected in boxes presented in the GPs practice. Nine opportunistic subjects, who were not a patient at one of the GPs practices mentioned above, heard about the study through one of the co-workers and offered to participate by filling out the brochure as well. Their questionnaires were analyzed together with the other questionnaires collected at the GPs practices mentioned above.

### Inclusion and exclusion criteria

In case two or more screening questions were answered positively, the ESS score was ≥ 10 and the patient had a BMI of ≥ 25, he or she met the predetermined inclusion criteria and was qualified to participate in the second part of the study. Acute interfering pathology, progressive cognitive impairment and important use of drugs with sleepiness, drowsiness or somnolence as known side effects, were set as exclusion criteria. Patients who had been taking low-dose sleep medication for several years were not excluded.

### Patient selection

Due to time limitations, only a limited number of patients who met the inclusion criteria could be offered to participate in the second part of the study, namely a home measurement using the Somnolter^®^. Eligible patients were contacted by phone to reassure they met the inclusion criteria and to offer them a home measurement to detect possible OSAHS. Patient files were analyzed and those patients with a higher risk for OSAHS (high ESS score, high BMI, comorbidities such as Down Syndrome, family members with sleep apnea,…) were contacted first. Nineteen of them gave their consent for the second part of the study.

### Home monitoring

A couple of hours before the patient went to bed, the device was attached to the patient by one of the co-workers of this study during a home visit. During this visit a more comprehensive personal history including risk factors, regular medication and family history, was taken (Additional file 1: Appendix 7). Patients were allowed to take their regular medications. The next morning, the device was retrieved and the patients were offered a questionnaire to ascertain whether they had encountered any side effects during the night (Additional file 1: Appendix 8).

### Somnolter^®^ device

**Figure 2:**
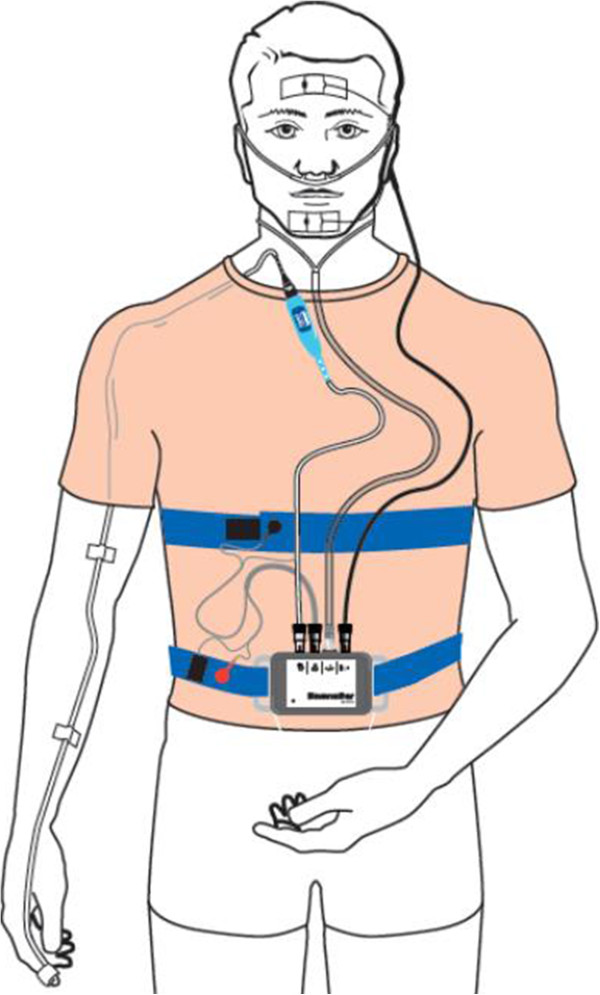
**The Somnolter Device.** Somnolter^®^recording the midsagittal jaw movements through sensors placed on the forehead and the chin, arterial oxygen saturation, body position, nasal airflow and thoracic movements. Adapted with permission from: http://www.nomics.be/uploads/pdf/Somnolter_Leaflet_EN-615.pdf.

Figure 2 is a representation of the Somnolter^®^ attachment. The Somnolter^®^ device records the following parameters: pulse-oximetry, body position, heart rate, nasal air flow and chest and abdominal movements. These are the traditional parameters measured during ambulatory sleep monitoring. The device additionally records mandibular movements: the Jaw Activity (Jawac) signal. Mandibular movements are said to be an excellent marker of respiratory effort during sleep, which is an important parameter in the diagnosis of sleep-disordered breathing (SDB) [20]. Furthermore, the recording of mandibular movements and its dedicated signal processing, allows to accurately assess the total sleeping time in a home setting [22]. This in contrast to a PSG in a hospital setting, camera recording is done to distinguish whether the patient is awake or asleep.

### Interpretation procedure

The interpretation of results was based on automatic analysis of the Somnolter^®^ and manual revision of the research team. The research team for example focused on the temporal relation of jaw movements and apneas/desaturations, which was not automatically analyzed by the Somnolter^®^. These movements (Figure 1), as mentioned before, represent the respiratory effort during sleep as they will show a period of increased amplitude and frequency reflecting the typical gasping mandibular movements occurring after an episode of apnea and desaturation. AASM criteria were used to distinguish between mild, moderate and severe OSAHS. Diagnosis of OSAHS was validated by two independent specialists in the near future.

### Approval

This study was approved by the Medical Ethics Committee of the Catholic University Hospital Leuven. All patients gave informed consent to the investigations. No physical or emotional harm was done to participating patients and all the data were processed and analyzed anonymously. There was no financial reward for those participating in this study.

## Results

**Figure 3:**
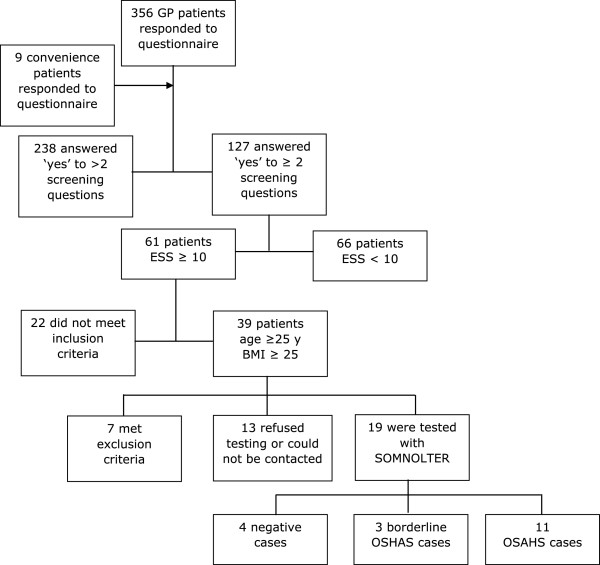
**Screening population algorithm.**

A total of 365 questionnaires were filled out by patients consulting the co-operating GPs practices or by patients who heard from the study by one of the co-workers. The mean age of this population was 53.67 ± 14.57 years old. The sex ratio (male/female) was 6/10. The mean ESS score was 6.56 ± 4.56 on a total of 24 points. A hundred and sixty-eight patients out of 365 responded positively to only one of the screening questions, 65 patients responded positively to two screening questions and 61 answered positively to all three questions (Figure 3). Of 127 patients with ≥ 2 positive screening questions, 66 patients had an ESS < 10 and 61 patients had an ESS ≥ 10.

Between the 61 patients of the eligible population, 32 patients also met the other inclusion criteria (age ≥ 25 and BMI ≥ 25) for diagnostic evaluation with Somnolter^®^. Seven of them were excluded based on the exclusion criteria mentioned earlier in this article. Nineteen subjects agreed to be tested by home-monitoring. Registration with Somnolter^®^ failed in one patient, resulting in a study population of 18 patients.

Patient characteristics of the study population are summarized in Table 1. The mean age of the study population was 53.06 years old and all of the patients were of Caucasian ethnicity. The sex ratio was 1/1. Of the 18 patients studied, 10 were treated with antihypertensive drugs. Five people had a history of a cardiovascular event, i.e. coronary stenting, cerebrovascular accident, transient ischemic attack, Lyme myocarditis and myocardial infarction. Eight subjects reported difficulties concentrating. Three were smoking at the time of this study and 3 had been smoking in the past. The average pack years of the smokers was 25 years. Almost all of the patients consumed alcohol on a regular basis, but there was no excessive alcohol use in any of the patients. Six of the 9 female patients were post-menopausal. Six patients had complaints of chronic rhinitis and/or sinusitis. At the time of examination, one person had a common cold.

**Table 1:** **Patients characteristics**

Patients	Sex	Age (y)	BMI (kg/m ^2^)	AHT	CVD	Memory / Concentration	Current smoker	Pack years	Alcohol (units/w)	Post-meno-pausal	History of recurrent sinusitis	Other important diagnosis	Family risk factors
**1**	F	57	25	No	No	Impaired	No	0	2	Yes	Yes	/	DM2
**2**	M	63	28	Treated	Coronal stents	Impaired	No	18	13	/	No	HyperCh	OSAHS
**3**	M	54	41	Treated	AMI and PAD	Normal	Yes	35	14	/	Yes	COPD	CV, DM2
**4**	F	48	40	Yes	Myocarditis	Impaired	Yes	30	4	No	No	/	OSAHS
**5**	F	47	25	Treated	No	Impaired	No	0	2	Yes	No	/	CV
**6**	F	56	27	No	No	Impaired	No	0	3	Yes	No	/	CV, DM2
**7**	M	38	31	No	No	Normal	No	0	3	/	Yes	DM2	OSAHS
**8**	F	78	30	Treated	No	Normal	No	0	1	Yes	Yes	Osteopor.	DM2
**9**	M	50	33	No	No	Normal	No	20	5	/	Yes	HyperCh	CV, DM2
**10**	M	60	30	Treated	CVA	Impaired	No	0	0	/	No	Epilepsy	CV
**11**	M	48	26	No	No	Normal	No	0	12	/	No	/	CV, DM2
**12**	F	28	29	No	No	Normal	No	0	1	No	Yes	/	OSAHS
**13**	M	67	32	Treated	AMI	Impaired	No	25	5	/	No	DM2	OSAHS
**14**	M	40	25	No	No	Normal	No	0	0	/	Yes	asthma	/
**15**	M	43	29	No	No	Normal	Yes	23	0	/	No	Down S/	CV, DM2
**16**	F	78	25	Treated	TIA	Impaired	No	0	0	Yes	No	CKD - TIA	/
**17**	F	51	34	Treated	No	Normal	No	0	4	No	No	/	OSAHS
**18**	F	62	35	No	No	Normal	No	0	2	Yes	Yes	HyperCh	CV

‘BMI’: Body Mass Index – ‘AHT’: arterial hypertension – ‘CVD’: cardiovascular disease - ‘Pack Years’: years smoked equal to 1 pack of cigarettes a day. – ‘Alcohol (units/w): amount of alcoholic units consumed on average in a week – ‘PAD’: Peripheral atherosclerotic disease – ‘CVA’: cerebrovascular accident – ‘COPD’: chronic obstructive pulmonary disease – ‘DM2”: Type 2 Diabetes mellitus – ‘EBV’: Epstein Barr virus – ‘AF’: Atrial fibrillation – ‘CDK’: Chronic kidney disease – ‘TIA’: Transient ischemic attack – ‘MVI’: Mitral Valve Insufficiency – ‘AMI’: Acute Myocardial Infarction – ‘HyperCh”: hypercholesterolemia – ‘osteopor’: osteoporosis – ‘Down S/’: Down Syndrome.

There appeared to be a heavy family burden in the study population. A family history of OSAHS, prior familial cardiovascular events and type 2 diabetes was reported by 6, 10 and 11 patients respectively.

Table 2 summarizes the most important information from the Somnolter^®^ reports. The mean ESS in our study population was 13.33 on a total of 24 points. No clear correlation between ESS and OSAHS was found. Except for one, all of the patients slept more than 5 hours. Two patients had an AHI < 5; this means no OSAHS according to the AASM criteria. Ten subjects had an AHI between 5 and 15, corresponding to mild OSAHS. Three patients had an AHI between 15 and 30, which is moderate OSAHS. Three subjects showed an AHI of ≥ 30, matching severe OSAHS.

**Table 2:** **Results questionnaires and Sonmolter** ^**©**^ **analysis**

Patients	Questions answered “positive”	ESS	Total sleep time	AHI	OAI	RERA I	RDI	RAI	SaO _2_ CT90	Oximetry Minimum 0 _2_ saturation	Diagnosis
1	3	12	7:18:19	14,5	0,4	3,1	17,7	12,3	0:02	77%	OSAHS (positional)
2	2	13	7:13:34	59,5	35,3	4,7	64,2	51,8	2:19	70%	Severe OSAHS
3	2	14	4:36:17	15,9	4,3	19,5	35,4	22,2	3:25	65%	Severe OSAHS
4	3	18	9:49:14	5,8	0,5	24,0	29,8	27,3	0:03	86%	Borderline OSAHS
5	3	18	7:49:30	4,1	0,9	4,1	8,2	6,6	0:00	90%	Normal pattern
6	3	16	7:06:09	7,5	1,3	6,8	14,2	12,0	0:03	86%	Normal pattern
7	2	11	7:50:56	14,7	3,3	15,2	29,8	25,0	0:07	79%	OSAHS
8	2	12	9:49:30	12,3	3,9	16,9	29,2	24,1	0:12	83%	OSAHS
9	2	10	5:25:53	21,4	10,7	15,1	36,5	29,8	0:10	78%	OSAHS
10	3	16	8:35:48	12,3	0,6	8,3	20,6	18,0	0:03	88%	Borderline OSAHS
11	3	14	7:35:31	12,0	0,8	2,6	14,6	8,4	0:03	86%	OSAHS
12	2	11	8:13:10	1,1	0,2	1,2	2,3	1,3	0:00:42	84%	Normal pattern
13	2	12	8:16:43	37,7	6,6	13,9	51,6	41,8	1:29	80%	Severe OSAHS
14	2	10	8:18:30	11,7	0	4,5	16,1	12,2	0:00	92%	Borderline OSAHS
15	2	10	9:01:43	60,8	46,5	12,7	73,5	62,1	4:18	67%	Severe OSAHS
16	3	18	9:56:27	12,7	2,9	11,3	23,9	20,2	0:02	84%	OSAHS
17	2	10	8:46:15	7,3	0,6	6,8	14,1	10,6	0:04	87%	Normal pattern
18	3	15	8:24:27	23,3	6,9	11,2	34,5	28,1	1:42	78%	OSAHS

Eight patients had an obstructive apnea index (OAI) ≤ 1 per hour, 6 had an OAI between 1 and 5 per hour, 1 person had an OAI between 5 and 10 per hour and the other 3 participants had a severe rise in OAI of 10.7, 35.3 and 46.5 respectively.

Other measured parameters were respiratory related arousal index (RERA I), respiratory disturbance index (RDI), respiratory arousal index (RAI) and the cumulative time below 90% oxygen saturation (SaO_2_ CT 90). Though these parameters varied a lot between the different patients as shown in Table 2, some form of association could still be seen. In patients with a SaO_2_ CT90 ≥ 1 hour (ranging from 1h29 minutes to 4h18 minutes), AHI, OAI, RDI and RAI increased equably with increasing SaO2 CT90 (Additional file 1: Appendix 9). This trend was not seen for RERA I. When SaO_2_ CT 90 was corrected for the total sleeping time, an even stronger trend could be seen (Additional file 1: Appendix 10).

Each patient was also questioned about the user friendliness of the Somnolter^®^. Results are summarized in Table 3. On average, the patients reported to have slept well. Three patients had the subjective feeling that they had slept less than usual. The Somnolter^®^ was not considered annoying during sleeping. Eight patients reported that one of the sensors disconnected during sleep. Most often the pulse-oximeter or the nasal cannula disconnected. This disconnection however had no consequences on the final analysis. As every sensor has an individual and unique import, there is only one import gate a sensor can be plugged into and there is only one way to connect them to the device so most of the patients were capable of reconnecting the sensors themselves whitin a few minutes. In the two cases where a sensor was disconnected for most of the time sleeping, the software could still analyze the data with the help of the multiple parameter recordings and the very effective Jawac signal. As in PSG, the multiple parameters recorded during sleep, will allow the software to appoint hypopnea and apnea events even when registration with one of the sensors has failed.

**Table 3:** **Patients’ experience**

Patient experience	Avg. score
Have you slept well?	2.7/4
How user friendly is the device?	3.0/4
Have you experienced the Somnolter^®^ as disturbing?	1.6/4
Technical problems	No
Loosened sensors	8
Score system : 0: not at all, 1: little, 2: neutral, 3: quite, 4: very much	

## Discussion

This pilot study revealed 11 new and 3 borderline cases of OSAHS on a total of 18 patients successfully tested with the Somnolter^®^. This suggests that the used case-finding strategy and questionnaire could adequately detect high risk patients. The implementation of the proposed strategy in the GPs practice is easy and little time-consuming. The three screening questions about snoring, daytime somnolence and difficulties to concentrate, can easily be embedded in a general risk determination. In this study, only the patients with ≥ 2 positive answers on the 3 screening questions were further questioned using the ESS. Further research is needed to see if it is also useful for patients with only 1 positive question to fill out the ESS. To determine if this proposed case-finding strategy increases the pretest probability in a statistically significant way, case control studies will be needed. These results also support the statement mentioned before, namely that a high percentage (up to 70-80%) of patients with OSAHS is undiagnosed in the general population. Some explanations are suggested. At first, GPs rarely think of OSAHS as a possible explanation for persisting complaints of fatigue. If no somatic explanation is found at first sight, the complaints are often attributed to mental causes such as depression or chronic fatigue syndrome. For instance, patient 15 received antidepressants from his GP to treat his daytime somnolence and lack of energy. The Somnolter^®^ showed that this patient had severe OSAHS, which could explain his symptoms. On the other hand depression might also be a consequence of OSAHS [17], as stated before. Secondly, when not handed out directly and systematically to patients, fewer brochures were filled out. This might reflect some kind of threshold for the patients to consult their GP about snoring, excessive daytime sleepiness or difficulties to concentrate during the day. This study specifically asked for these symptoms through 3 screening questions. A final explanation for this high number of undetected OSAHS is the fact that in-hospital PSG appears to discourage patients. In the future the Somnolter^®^ might be a feasible alternative for the current PSG in the hospital, possibly resulting in a higher number of OSAHS diagnosis. When the diagnosis of OSAHS is confirmed, patients would only need one admission to test and regulate the pressure of the CPAP-device. By reducing the need for admission, waiting times for overnight PSG as well as health care costs can be reduced.

Besides the 14 new diagnoses of OSAHS, other interesting data regarding the sleep quality of patients were analyzed by the Somnolter^®^. This allowed the authors to draw supplementary conclusions. First, for some patients the authors assigned another degree of OSAHS than would be given by the AASM. Patient 1 for example would normally be diagnosed as having mild OSAHS, but because of a very low OAI (0.4/h) and a SaO_2_ CT90 of only 2 minutes, both related to supine position, she was finally diagnosed with positional OSAHS. Although an AHI of only 15.9/h, patient 3 was considered to have severe instead of moderate OSAHS and this because of long and deep desaturations (minimum SaO_2_ of 65%) and a SaO_2_ CT90 of more than 3 hours. These 2 cases indicate that besides AHI other parameters such as OAI, body position, SaO_2_ CT90, maximum desaturation etc. should be considered when assigning the degree of OSAHS.

Secondly, the Somnolter^®^ allows to distinguish the more severe cases of OSAHS and to make a suggestion towards an appropriate treatment. In patient 15 the SaO_2_ CT90 was no less than 4 hours and 18 minutes with a minimum SaO_2_ of 67%. This patient for example has very severe OSAHS and should undoubtedly be referred for further treatment. It is remarkable that this patient, the most severe case of OSAHS in this study population, is a patient with Down syndrome. As mentioned before, Down syndrome significantly increases the risk of OSAHS [23]. On the other hand, in patient 1 the apneas were related to supine position. Taking measures to prevent this patient from sleeping in supine position might be sufficient to minimalize the apneas and deep desaturations and to decrease symptoms.

Finally, in patients with a SaO_2_ CT90 ≥ 1 hour (ranging from 1h29 minutes to 4h18 minutes), AHI, OAI, RDI and RAI increased equably with increasing SaO_2_ CT90. When SaO_2_ CT 90 was corrected for the total sleeping, an even stronger trend could be seen. Further research is needed to establish a significant correlation.

The Somnolter^®^ is a new generation device since the Jawac sensors analyze jaw movements. The Jawac sensors are a powerful tool giving an image of a persons’ snoring pattern as well as gasping movements during sleep. Figure 1 shows gasping movements typically appearing after a period of apnea and desaturation, and followed by a slow increase of SaO_2_. In the future, fewer sensors might be sufficient to adequately diagnose OSAHS. For instance the Jawac sensor to detect gasping combined with a pulse oxygen meter to detect desaturations. Devices measuring only these two parameters are already developed but further research is needed to confirm them as an adequate diagnostic tool.

It is important to stress that the questionnaire used in this study cannot replace a thorough history taking. For example, one of the patients was in a mourning phase which could cause her troubled sleep and explain her complaints of fatigue. This was only known after testing with the Somnolter^®^. Another patient also reported, after testing, that her fatigue could possibly be explained by her recent motherhood. The used questionnaire did not pick up these causes of fatigue. Therefore, an extra question might need to be added to the questionnaire. For example: *‘Do you have any idea why you are extremely tired?’*.

## Limitations

The authors are well aware of the limitations of this pilot study. In 3 of the 5 general practices, the brochure could not be distributed in a systematic way. As a consequence, in these general practices only prior interested individuals filled out the document, resulting in a possible selection bias. In two GPs practices, the brochures and questionnaires were handed out systematically by a secretary, with very high response rates. Because of this variable approach, the data cannot be considered to be of epidemiologic value. Another limitation of the brochures was an incomplete or incorrect completion by the patient. Because of the short time period during which this pilot study took place, in addition to having only one Somnolter^®^-device available, a limited number of patients could be tested.

## Conclusion

This pilot study set up a case-finding strategy to identify high risk patients in whom it could be useful to screen for OSAHS. Although the proposed strategy still needs to be optimized, the authors concluded that their strategy could definitely be helpful in selecting high risk patients. They detected OSAHS in 14 out of 18 patients tested with the Somnolter^®^, suggesting a high percentage of undiagnosed OSAHS in the general population.

In the future the Somnolter^®^, already proven to have a similar sensitivity and specificity as the gold standard in-hospital polysomnography, might be a feasible alternative to diagnose OSAHS. Patients experience the Somnolter^®^ as less discouraging than polysomnography since it is more comfortable and can be done in a home-setting.

## Electronic supplementary material

Additional file 1: **Appendix 1-10 [** [24]**-** [27]**].** (DOCX 389 KB)

## Abbreviations

PSG: Polysomnography
OSAS: Obstructive sleep apnea syndrome
OSAHS: Obstructive sleep apnea hypopnea syndrome
CHAHS: Central sleep apnea-hypopnea syndrome
UARS: Upper airway resistance syndrome
AASM: American Academy of Sleep Medicine
ESS: Epworth sleepiness scale
OAI: Obstructive apnea index
AHI: Apnea/Hypopnea index
SDB: Sleep-disordered breathing
GP: General practitioner
JAWAC: Jaw activity
RERA I: Respiratory related arousal index
RDI: Respiratory disturbance index
RAI: Respiratory arousal index.
