# Supplementary material for: Detection of sleep apnea by case-finding and home monitoring with Somnolter®: a pilot study
Source: BMC Res Notes. 2014 Sep 8;7:616. doi: 10.1186/1756-0500-7-616 (PMC4174633; doi:10.1186/1756-0500-7-616)
Supplement: Supplementary file 1 — Additional file 1: Appendix 1-10 [ [24]- [27]]. (DOCX 389 KB) [file 13104_2013_3172_MOESM1_ESM.docx]

**Additional file**

**Abbreviations**

OSA: Obstructive sleep apnea syndrome

AHI: apnea-hypopnea index

BMI: body mass index

REM: rapid eye movement

CV: cardiovascular

CVD: cardiovascular diseases

BP: blood pressure

PSG: polysomnography

WSCS: Wisconsin Sleep Cohort Study

SHHS: Sleep Heart Health Study

SaO_2_: oxygen saturation

IHD: ischemic heart disease

CAD: coronary artery disease

**Appendix 1: Prevalence data of OSAHS**

| **Table 1 : Prevalence data of OSAHS** | | | |
| --- | --- | --- | --- |
| Study | **Population** | **Method** | **Results** |
| Young et al; 1993 [6] | Adult Caucasian Population  602 middle-aged adults | Polysomnography  Cut off AHI = 5 | AHI >5: 24% in men, 9% in women  OSAHS: 4% in men, 2% in women |
| Bixler et al; 2001 [7] | Adult Caucasian Population  1000 women, 741 men | Polysomnography  cut off AHI = 10 | AHI >10: 4% in men, 2% in women |
| Hrubos-StrØm et al; 2011 [8] | Norwegian adults  16,302 (BQ), 518 (PSG) | Berlin Questionnaire and Polysomnography | AHI >5: 16% (male 21%, female 13%) |
| Fuhrman et al; 2012 [9] | 12,203 French adults | Witnessed apneas, snoring and daytime excessive sleepiness | ESS >10: 4,9% |
| Hiestand et al; 2006 [10] | 1,506 American adults | Berlin Questionnaire | High risk OSA: 26% (31% men, 21% women) |
| Lam et al; 2007 [2] | Asian population | Literature study of articles between 1993 and 2004 | Prevalence 4.1-7.5% in men, 2.1-3.2% in women |
| Marcus et al; 2013 [13] | Pediatric population | Literature study of articles between 1999 and 2011 | Prevalence 1.2%-5.7% |

**Appendix 2: AASM Diagnostic criteria of OSAHS and definitions (American Academy of Sleep Medicine)**

| **Diagnostic criteria of OSAHS and definitions; adapted from [12]** | | |
| --- | --- | --- |
| Any individual must fulfill criterion A or B, plus criterion C to be diagnosed as having OSAHS. | | |
| A | Excessive daytime sleepiness that cannot be explained by other factors | |
| B | Two or more of the following, not explained by other factors   - Choking or gasping during sleep - Recurrent awakenings from sleep - Unrefreshing sleep - Daytime fatigue - Impaired concentration | |
| C | Overnight monitoring demonstrates 5 or more obstructed breathing events per hour during sleep. These may include any combination of AHI or RERA, as defined below. | |
| Definitions | | |
| Apnea/hypopnea index (AHI) | | 1. A clear decrease (>50%) from baseline in the amplitude of a validated measure of breathing during sleep.  2. A clear amplitude reduction of a validated measure of breathing during sleep that does not reach the above criterion but is associated with either an oxygen desaturation of >3% or an arousal.  3. The event lasts 10 seconds or longer ^(appendix 1)^. |
| Respiratory effort-related arousal (RERA) | | A sequence of breaths characterized by increasing respiratory effort leading to an arousal from sleep, but which does not meet criteria for an apnea or hypopnea. These events must fulfill both of the following criteria:  1. Pattern of progressively more negative esophageal pressure, terminated by a sudden change in pressure to a less negative level and an arousal  2. The event lasts 10 seconds or longer ^( appendix 1)^. |

**Appendix 3: The purview and limitations of the recommendations for OSAHS definition according to the American Academy of Sleep Medicine**

To understand the purview and limitations of these criteria, it is important to know how the cut-off points were determined. The use of an event frequency of five per hour as a minimal threshold value for elevated AHI, was based on epidemiological data. These data show that from an AHI > 5, minimal effects on health occur. For example, patients with an AHI > 5, more often suffer from hypertension, sleepiness or were involved in motor vehicle accidents more frequently than those with an AHI < 5 [24, 25].

Additionally, limited data from intervention studies suggest that treatment with CPAP was associated with improvements in vitality, mood, and fatigue in subjects with an AHI between 5 and 30, and improvements in sleepiness and neurocognitive function in subjects with AHI levels of 5 to 15 [26,27].

The '10 second' criterion is used by convention. The task force recognized that although there was no absolute justification for this cutoff, it is a standard that has been used since the first description of sleep apnea, it is what all current research and clinical studies use, and there is no data available indicating that a different criterion is superior [12].

**Appendix 4: Modifiable and non-modifiable risk factors for OSAHS**

| **Modifiable and non-modifiable risk factors associated with OSAHS** | |
| --- | --- |
| Modifiable Risk Factors for OSA | **Non-Modifiable Risk Factors for OSA** |
| Obesity Smoking Drugs (opiates, benzodiazepines, alcohol) Nasal congestion or obstruction Menopause | Gender: men > women Genetic predisposition Ethnicity Aging Cranial abnormalities |

**Appendix 5: Morbidity and mortality associated with OSAHS**

| **Morbidity and mortality associated with OSAHS [16-18]** | |
| --- | --- |
| Morbidity and mortality | **Strength of evidence** |
| Death from all-causes Cardiovascular diseases  Heart failure  Arrhytmias  Arterial hypertension  Coronary artery disease  Stroke  Cardiovascular mortality  Diabetes  Depression | Independent risk factor in men> women  suggested impact of OSAHS on day time left ventricular systolic function  Suggested association between OSAHS and atrial fibrillation  Independent risk factor, dose dependent response in hypertension with worsening sleep related breathing measures across all ages and ethnic groups  No consistent link, but definitely a potential association in men  OSAHS is an independent risk factor men>women in a dose-dependent way, reciprocal association  OSAHS is an independent risk factor  Suggested association, further research necessary  Suggested association, further research necessary |

**Appendix 6:** **Underlying pathophysiological mechanisms of OSAHS**

**Underlying pathophysiological mechanisms of OSAHS** (adapted from [15,16 ] )


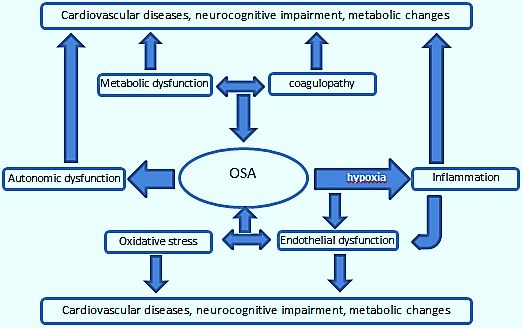


**Appendix 7: Overview of the comprehensive personal history, including risk factors regular medication, and family history taken during home visit.**

**Personal information**

| **Name and surname** |  | **Sex:** |  |
| --- | --- | --- | --- |
| **Date of birth** |  | | |

**Anamnestic information**

| *A. Associated morbidity?* | yes | No | Not know |
| --- | --- | --- | --- |
| 1. Arterial hypertension: (systolisc blood pressure ≥ 140 mmHg,  diastolic bloodpressure ≥90 mmHg) |  |  |  |
| 2. Previous cardiovascular diseases  If so, can you name these? |  |  |  |
| 3. Defective memory/difficulties to concentrate? |  |  |  |
| 4. Overweight or obesity (BMI > 27)  If so, BMI? |  |  |  |
| 5. Nicotine use  If so, how many cigarettes a day?  Number of pack years? |  |  |  |
| 6. Alcohol use  If so, how many units per week? |  |  |  |
| 7. Age > 30 years |  |  |  |
| 9. Women: postmenopausal? |  |  |  |
| 10. Symptoms of chronic sinusitis/rhinitis? |  |  |  |
| 11. Diabetes mellitus type 2 |  |  |  |

| *B. Other important morbidities?* |
| --- |
|  |
|  |
|  |
|  |
|  |
|  |
|  |
|  |
|  |
|  |

| *E. Family History* | Severity? | Which family member is affected? |
| --- | --- | --- |
| Sleep apnea |  |  |
| Cardiovascular disease |  |  |
| Diabetes mellitus (type2) |  |  |
| Other |  |  |

***C. Does the patient have a common cold at this moment?***

***D. Ethnicity:***

| *F: Clinical examination* | | |
| --- | --- | --- |
| Weight |  | kg |
| Length |  | cm |
| Neck circumference |  | cm |
| Waste circumference |  | cm |
| Hip circumference |  | cm |
| Systolic bloodpressure |  | mmHg |
| Diastolic bloodpressure |  | mmHg |
| Lung auscultation |  |  |
| Heart auscultation |  |  |

| *H: Additional information* |
| --- |
|  |
|  |
|  |
|  |
|  |
|  |

| *G: Current medication* | *Dose* | *Frequency/dag* | *Time* |
| --- | --- | --- | --- |
|  |  |  |  |
|  |  |  |  |
|  |  |  |  |
|  |  |  |  |
|  |  |  |  |
|  |  |  |  |
|  |  |  |  |

**Appendix 8: Questionnaire testing the user friendliness of Somnolter®**

***
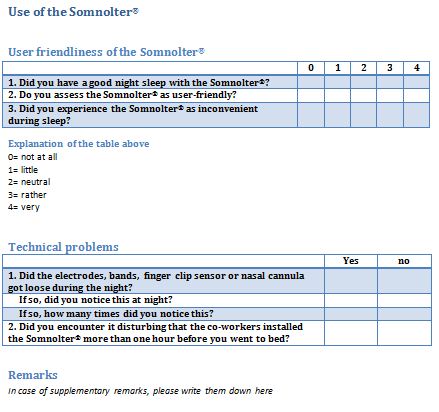
***

**Appendix 9: Association between SaO_2_ and AHI, OAI, RDI and RAI**

**
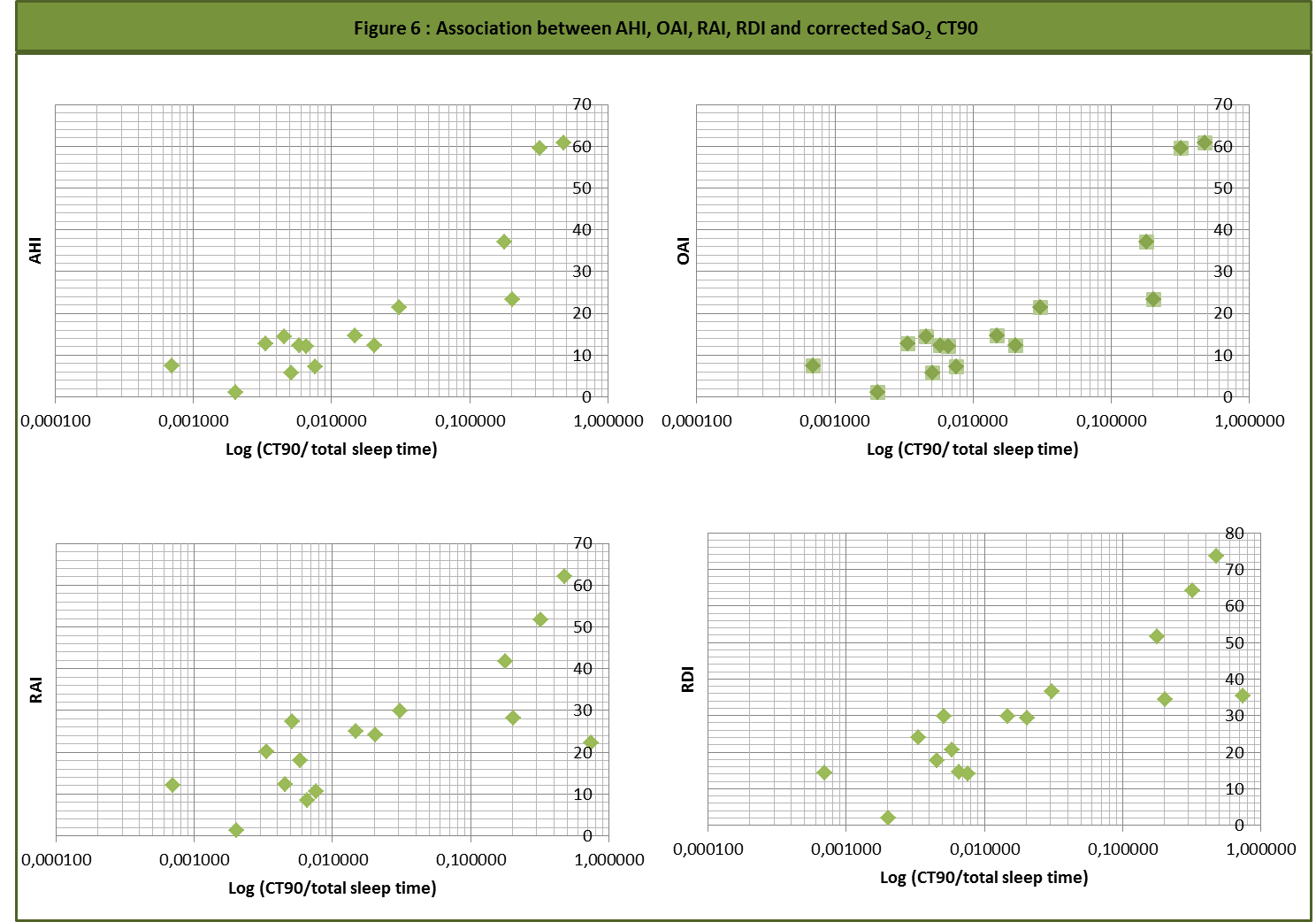
**

**Appendix 10: Association between SaO_2_ CT90 and AHI, OAI, RDI and RAI**

**
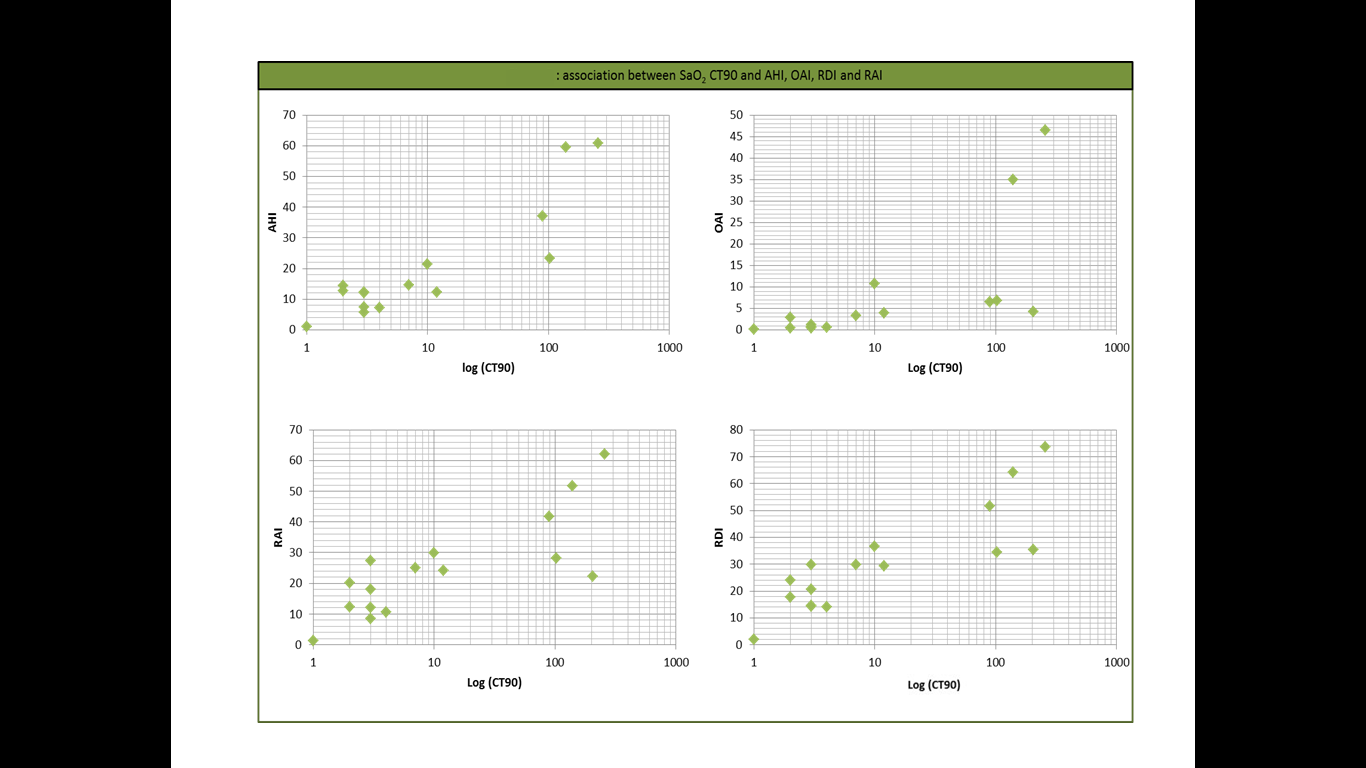
**
